# Modulation of the rat intestinal microbiota in the course of *Anisakis pegreffii* infection

**DOI:** 10.3389/fvets.2024.1403920

**Published:** 2024-05-09

**Authors:** Min-hao Zeng, Shan Li, Qing-bo Lv, Xiao-xu Wang, Abdul Qadeer, Mohamed H. Mahmoud

**Affiliations:** ^1^School of Biotechnology, Jiangsu University of Science and Technology, Zhenjiang, China; ^2^Jiangxi Provincial Key Laboratory of Cell Precision Therapy, School of Basic Medical Sciences, Jiujiang University, Jiujiang, China; ^3^Key Laboratory of Zoonosis Research, Institute of Zoonosis, College of Veterinary Medicine, Ministry of Education, Jilin University, Changchun, China; ^4^Department of Cell Biology, School of Life Sciences, Central South University, Changsha, China; ^5^Department of Biochemistry, College of Science, King Saud University, Riyadh, Saudi Arabia

**Keywords:** *Anisakis pegreffii*, rat, microbiota, diversity analysis, host effects

## Abstract

**Background:**

*Anisakis* are globally distributed, marine parasitic nematodes that can cause human health problems, including symptoms such as vomiting, acute diarrhea, and allergic reactions. As parasitic nematodes that primarily affect the patient’s digestive tract, intestinal helminths can interact directly with the host microbiota through physical contact, chemicals, or nutrient competition. It is widely accepted that the host microbiota plays a crucial role in the regulation of immunity.

**Materials and methods:**

Nematodes collected from the abdominal cavity of marine fish were identified by molecular biology and live worms were artificially infected in rats. Infection was determined by indirect ELISA based on rat serum and worm extraction. Feces were collected for 16S rDNA-based analysis of microbiota diversity.

**Results:**

Molecular biology identification based on ITS sequences identified the collected nematodes as *A. pegreffii*. The success of the artificial infection was determined by indirect ELISA based on serum and worm extraction from artificially infected rats. Microbiota diversity analysis showed that a total of 773 ASVs were generated, and PCoA showed that the infected group was differentiated from the control group. The control group contained five characterized genera (*Prevotellaceae* NK3B31 group, *Turicibacter*, *Clostridium sensu stricto* 1, *Candidatus Stoquefichus*, *Lachnospira*) and the infected group contained nine characterized genera (*Rodentibacter*, *Christensenella*, *Dubosiella*, *Streptococcus*, *Anaeroplasma*, *Lactococcus*, *Papillibacter*, *Desulfovibrio*, *Roseburia*). Based on the Wilcoxon test, four processes were found to be significant: bacterial secretion system, bacterial invasion of epithelial cells, bacterial chemotaxis, and ABC transporters.

**Conclusion:**

This study is the first to analyze the diversity of the intestinal microbiota of rats infected with *A. pegreffii* and to determine the damage and regulation of metabolism and immunity caused by the infection in the rat gut. The findings provide a basis for further research on host-helminth-microbe correlationships.

## 1 Introduction

Anisakiasis is a parasitic disease caused by the ingestion of larvae of the Anisakis nematode. These larvae are commonly found in marine fish worldwide. Consumption of raw or undercooked fish poses a risk of infection due to the presence of Anisakis larvae within the fish tissue (1). Symptoms of Anisakiasis typically include gastrointestinal disturbances such as vomiting, nausea and diarrhea (2). Additionally, allergic reactions and ectopic parasitism may occur due to the migration of the nematode larvae, presenting a potential health threat (3–6). The infection is prevalent globally and is particularly associated with regions where raw or lightly processed seafood is commonly consumed, such as Japan, Korea, China, Taiwan, Portugal, and Chile (7). In 2010, the European Food Safety Authority (EFSA) estimated that there were around 20,000 reported cases of Anisakiasis worldwide. In Japan alone, the average annual incidence of Anisakiasis in 2018–2019 was 19,737, based on insurance claims records (8, 9). Anisakiasis is widely prevalent in seas around the globe, have been identified by the FAO (Food and Agriculture Organization of the United Nations) as one of the 10 most important parasites affecting humans and fish (10).

As parasitic nematodes that primarily affect the patient’s digestive tract, intestinal helminths can interact directly with the host microbiota through physical contact, chemical compounds, or nutritional competition. It is widely recognized that the host microflora plays a crucial role in regulating immunity (11–13). Parasites can also indirectly impact immunity and other physiological processes by regulating the host gut microbiota (14). Changes in gut microbes can also protect the host from parasites (15). Research has demonstrated that *Haemonchus contortus* has a significant impact on the gastrointestinal microbiota in lambs. It also reduces the α-diversity of the gastrointestinal microbial community in the rumen, abomasum, and duodenum, and disrupts the partitioning of amino acids for protein digestion and absorption (16). *Ascaris lumbricoides*, belonging to the superfamily Ascaridoidea along with Anisakis, is also capable of reducing the microbiota diversity of the porcine gut and altering the metabolic potential of the host (17).

It is of great interest to investigate whether Anisakiasis induces changes in the host gut microbiota. Using 16S rRNA sequence-based NGS sequencing and bioinformatics analysis, we researched the response of rat fecal microbiota communities to Anisakiasis to gain insight into how *A. pegreffii* affects host gut microbiota and metabolism. This statement provides a basis for further studies on the interactions between *Anisakis*-hosts-microbiota.

## 2 Materials and methods

### 2.1 Parasites collection

We obtained fresh, unfrozen marine fish such as Little Yellow Croaker (*Larimichthys polyactis*) and Largehead hairtail (*Trichiurus lepturus*) from markets in Shanghai City, China. The fish were dissected using scissors to expose the organs and digestive tract. Nematodes were isolated from tissues including the abdominal cavity, stomach, intestines, gonads, and muscles using forceps or needles. The worms were then washed three times with saline solution to remove any attached fish tissue.

### 2.2 Identification of parasites

The nematodes were categorized by microscopic observation and genomic DNA was extracted from randomly selected worms for molecular identification. In brief, the nematodes were homogenized in 200 μL of lysis buffer (Tris–HCl 100 mM, EDTA 25 mM, NaCl 500 mM, Protease K 10 μL), then incubated at 56°C for 6 h. After centrifugation at 12,000 rpm for 5 min to remove the precipitate, 200uL of Tris-saturated phenol: chloroform: isoamyl alcohol mixture (25, 24, 1, pH 8.0) were added, and then centrifuged at 12,000 rpm for another 5 min. The supernatant aqueous phase was transferred to a new tube. To extract genomic DNA, 200 μL of pre-cooled anhydrous ethanol was added to the sample, followed by thorough mixing. The mixture was then centrifuged at 12,000 rpm for 10 min, and the supernatant was carefully removed, leaving the precipitate behind. Wash the precipitate twice with 200 μL of 75% ethanol. Finally, dissolve the precipitate with ddH_2_O. Genomic DNA was used as the template for PCR. The molecular identification by PCR was based on the ITS (Internal Transcribed Spacer) rDNA regions, the sequences were amplified using NC5 (5′- GTA GGT GAA CCT GCG GAA GGA TCA TT −3′) and NC2 (5′- TTA GTT TCT TTT CCT CCG CT −3′) primers (18, 19). PCR reaction (20 μL) contained 10 μL 2 × Hieff PCR Master Mix (Yeasen, Shanghai, China), 0.2 μL of each 10 μM NC5 and NC2 primers, ddH_2_O up to 20 min were performed on the following conditions: 95°C for 3 min (initial denaturation), then 35 cycles of 95°C for 15 s (denaturation), 55°C for 15 s (annealing), and 72°C for 1 min (extension), and a final extension at 72°C for 5 min. The PCR products were analyzed by PCR-RFLP using *Hinf* I endonuclease (NEB, Beijing, China) and the PCR products were sequenced (20). For the elongation factor (EF1 α-1 nDNA) nuclear gene, the sequences were amplified using EF-F (5′- TCC TCA AGC GTT GTT ATC TGT T − 3′) and EF-R (5′- AGT TTT GCC ACT AGC GGT TCC -3′). The PCR reaction was essentially the same as above, but with an annealing temperature of 58°C (21). After sequencing, the obtained sequences and peak maps were aligned with the reference of EF1 α-1 nDNA sequences using SnapGene software 4.2.4^1^.

### 2.3 Artificial infection of rats

With reference to the methodology of previous studies, a total of 6 5-6-week-old Sprague–Dawley (SD) rats were artificially infected with 10 live *A. pegreffii* using the infant rectal drug delivery tubes (22). Briefly, active worms are inserted into the tube stretching from the mouth of the tubes, the tubes were inserted into the rats’ stomach, and a syringe was used to push the worms into the stomach. Six additional rats were used as negative controls. Serum samples were collected at the 1, 2, 3, and 5 weeks after infection, and feces were collected at 5 weeks after infection. The collected samples were either stored at −80°C or directly used in additional experiments. Five *A. pegreffii* were homogenized in pre-cold PBS (Phosphate Buffered Saline). The precipitate was removed by centrifugation, and the supernatant protein solution was filtered using a 0.22 μm filter. The concentration was determined using a BCA kit (Beyotime, Shanghai, China). An indirect ELISA was performed using worm extraction proteins and rat serum to diagnose the success of the artificial infection. Briefly, 96-well microtiter plates were coated with 1 μg of worm extraction protein in 100 μL of carbonate buffer (150 mM Na2CO3, 349 mM NaHCO3, pH 9.6) and incubated at 4°C overnight. After washing three times, the plates were blocked with 5% bovine serum albumin (BSA) for 2 h at 37°C. Sbsequently, rat sera diluted in PBST at 1:400 were added for 2 h at 37°C. HRP-conjugated goat anti-rat IgG antibody (Servicebio, Wuhan, China) was used as a secondary antibody at a 1:5000 dilution, and the reaction were revealed by TMB (Beyotime, Shanghai, China) for 15 min at 37°C and stopped by 2 M H2SO4. The reaction was measured at 450 nm with an ELISA reader. Data are represented as the mean ± standard deviation (SD), and the *t*-test was used for comparisons between the two groups. GraphPad Prism 8.3.1 (GraphPad Software, Inc., San Diego, CA, United States) was utilized for statistical analysis and generating graphs. *p*-values <0.05 were considered statistically significant.

### 2.4 Analysis of 16S rRNA gene sequences

The extraction of fecal genomic DNA and the 16S amplification were both provided by Wuhan Wanmo Technology Co., Ltd. The 16S rDNA V3-V4 region of the sample DNA was amplified using common primers 341-F (5′- CCT AYG GGR BGC ASC AG −3′) and 806-R (5′- GGA CTA CNN GGG TAT CTA AT −3′), and the resulting products were used to construct the libraries (21), which were then sequenced using the BGISEQ-500 platform. Poor quality bases of the paired-end sequence data were trimmed using Trimmomatic v0.36 (23) and were merged using FLASH v1.20 (24). The sequences were then imported into QIIME2 (25). Species annotation was performed on the obtained Amplicon Sequence Variants (ASVs) based on the 16S bacterial ribosomal databases SILVA (Release 128) (26), RDP (v1.6) (27), and Greengene (Release 13.5) (28), and the number of annotations and scores were counted at the phylum, family, and genus levels, and beta diversity was analyzed by PCoA based on Bray-Curtis and Jaccard distance. Species differing between groups were screened using Linear discriminant analysis Effect Size (LEfSe), (LDA > 2 as the threshold). The results of the analysis were visualized through the online tool TUTOOL^2^.

## 3 Results

### 3.1 Diagnosis for *Anisakis pegreffii* infection in rats

**Figure 1:**
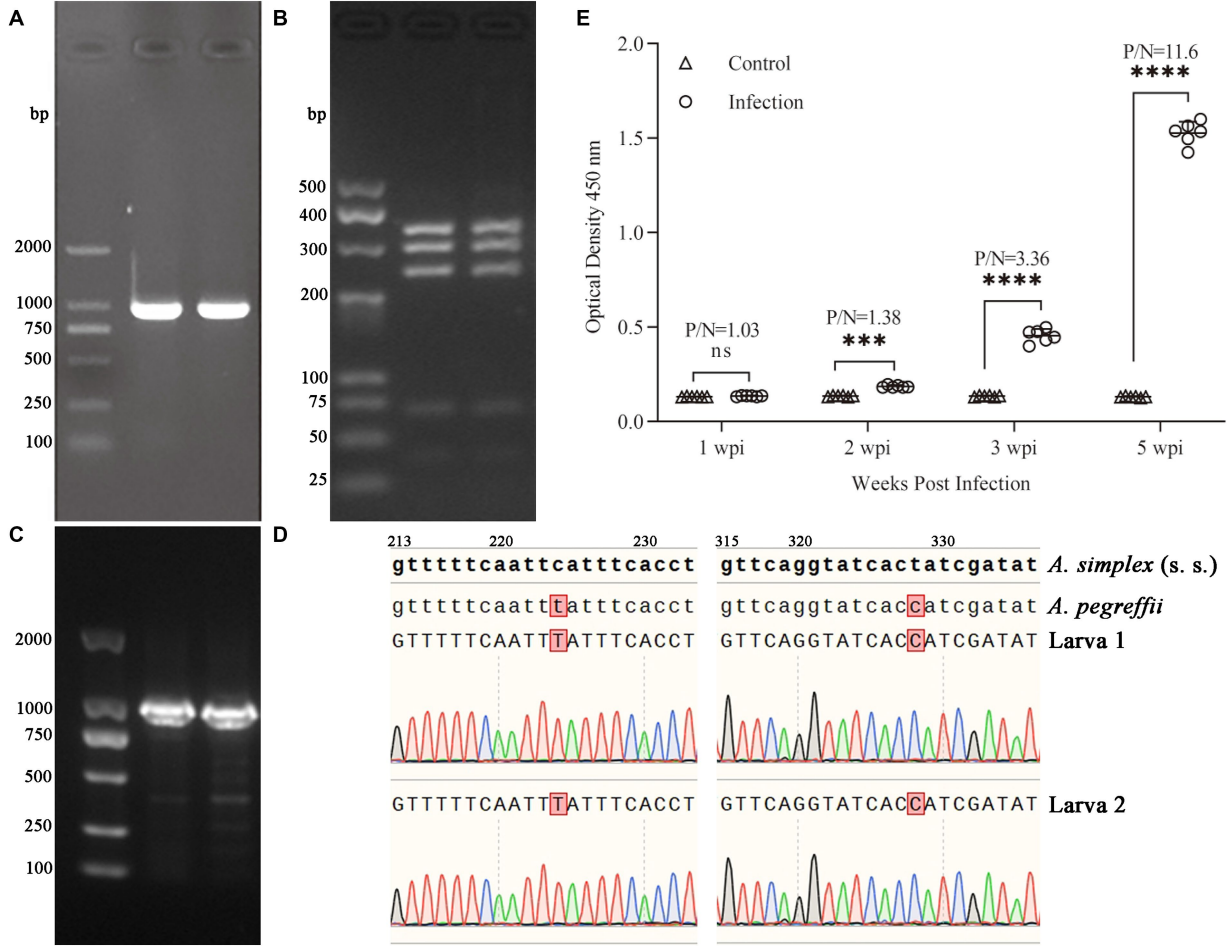
Electrophoresis of PCR products for ITS sequences in 1.0% agarose gel **(A)** and PCR-RFLP in 3.5% agarose gel **(B)** of *A. pegreffii*. The fragment sizes were all as expected. Results of electropherogram in 1.0% agarose gel **(C)** and alignment **(D)** of EF1 α-1 sequences. Indirect ELISA results **(E)** showed that OD450 of the infected group was significantly higher than that of the control group, and the P/N (Positive/Negative) values had reached 3.36 at 3 wpi, indicating that the rats were successfully infected with *A. pegreffii*.

The ITS fragment (approximately 1,000 bp) from each sample was amplified (Figure 1A). The PCR-RFLP showed three sharp fragments of 370, 300 and 250 bp consistent with *A. pegreffii* (Figure 1B). After sequencing, the target sequence showed 99.5% identity with *A. simplex* and up to 100% identity with *A. pegreffii* (GenBank Accession: AY821739.1 and MF820020.1, in Supplementary Figure S1). Based on the EF1 α-1 sequence of *A. simplex* (s. s.) and *A. pereffii* (GenBank Accession: KP326558.1 and MH443145.1), the sequences of the larvae obtained in this study were consistent with *A. pegreffii* and did not exist overlapping peaks (Figures 1C,D). Therefore, based on the detection method we used, the larvae used in this study were preliminarily identified as *A. pegreffii*. Based on the diagnostic results of the indirect ELISA assay, the results of all rat sera in the infection group were significantly higher than those of the control group at 2 weeks post-infection (wpi). As the duration of infection increased, the optical density 450 nm and P/N (Positive group/Negative group) values of the ELISA results for the infection group also increased significantly. At 5 wpi, the mean P/N values for both the infection and control groups were as high as 11.6 (Figure 1E). Therefore, the rats in the infected group were diagnosed with *A. pegreffii* infection, while those in the negative group were not infected.

### 3.2 *Anisakis pegreffii* restructure microbiota community in rats

**Figure 2:**
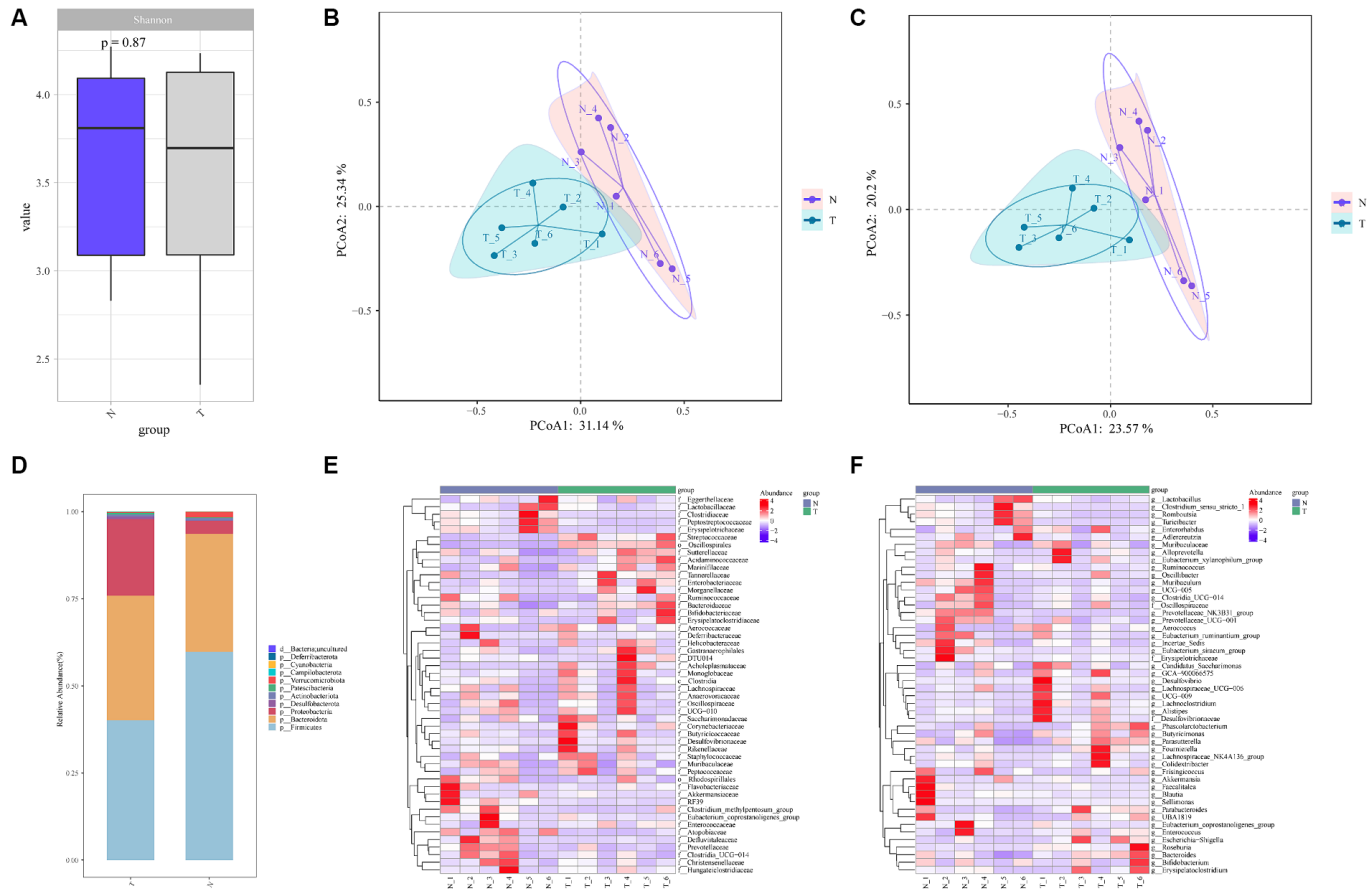
Alpha and beta diversity analyses between feces microbiota of *A. pegreffii* infection rats and control rats. N-negative control samples, T-infection samples. **(A)** Shannon Index Box Chart indicated no significant differences in alpha diversity. Beta-diversity analyses of clustering profiles based on Bray-Curtis **(B)** and Jaccard **(C)** showed the existence of significantly differences in beta diversity. **(D)** Bar plot of species composition at Phylum level. **(E)** Family level heatmap maps. **(F)** Genus-level heatmap maps.

A total of 773 ASVs were generated. The number of ASVs increased gradually with the sequencing depth, which met the analysis requirements (Supplementary Figure S2A). The relative abundance curves of the top 200 ASVs were smooth, indicating an even distribution of ASVs (Supplementary Figure S2B). At more than 3 biological replicates in each of the infection and control groups, the cumulative species curve tended to flatten, and the number of species did not significantly change with additional replicates. The number of replicates met the analysis requirements (Supplementary Figure S2C). Alpha diversity was assessed using Shannon’s index at various levels. The results indicated no significant differences in diversity (Figure 2A). However, beta-diversity analyses revealed a significant separation of clustering profiles of the PCoA indices based on Bray-Curtis and Jaccard (Figures 2B,C). This suggests that *A. pegreffii* infections have a significant impact on the composition of the gut microbiota.

A total of 11 Phylum were obtained from the annotation of the two groups, with roughly the same composition in both groups (Figure 2D). Among them, Firmicutes and Bacteroidota were the dominant Phylum in both groups. In the control group, 61.93% of the species belonged to Firmicutes, with an abundance of 59.75, and 29.66% of the species belonged to Bacteroidota, with an abundance of 33.89%. In the infected group, 58.82% of the species belonged to the Firmicutes Phylum with an abundance of 40.15 and 32.86% of the species belonged to the Anamorph Phylum with an abundance of 35.76%. The abundance of the Proteobacteria Phylum was higher in the infected group compared to the control group. The top 50 Families and Genera in terms of abundance were selected and analyzed at the Family and Genus level, respectively, and it was evident that there were significant changes between the infected group and the control group at the Family and Genus level (Figures 2E,F).

**Figure 3:**
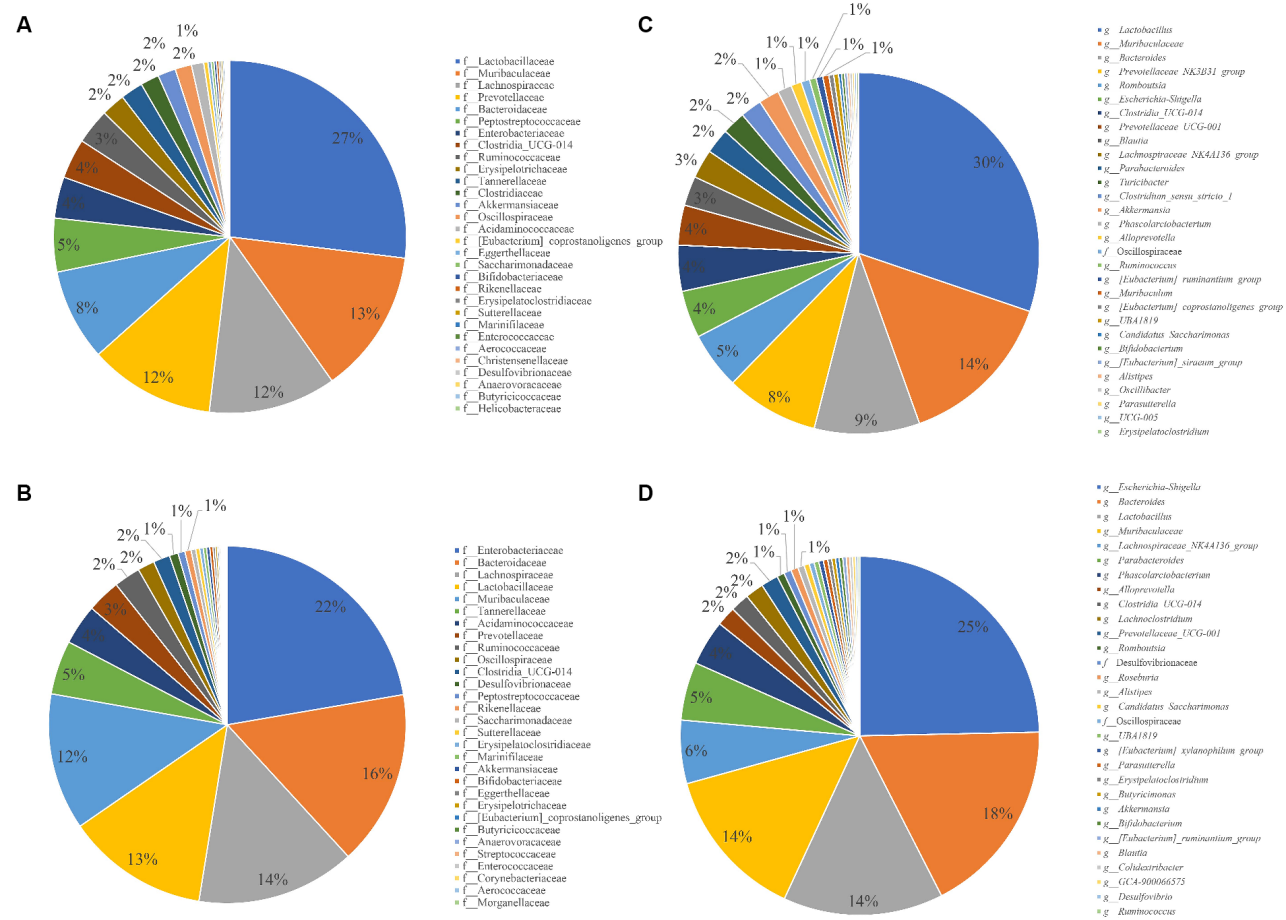
Pie chart for the proportion of species in different groups and taxonomic levels. **(A)** Composition of gut microbiota at family level in control group. **(B)** Composition of gut microbiota at family level in Infection group. **(C)** Control group at the Genus level. **(D)** Infection group at the Genus level.

The relative abundance of the top 30 families and genera in each group was statistically analyzed (Figures 3A,B). The families with more than 10% abundance in the control group were Lactobacillaceae, Lachnospiraceae and Prevotellaceae, and the families with more than 10% abundance in the infected group were Enterobacteriaceae, Bacteroidaceae, Lachnospiraceae, Lactobacillaceae and Muribaculaceae; compared to the control group, the relative abundance of Enterobacteriaceae in the infected group increased by 18.4%, Bacteroidaceae by 7.42%, Lactobacillaceae by 14.05%, Prevotellaceae by 8.46% and Peptostreptococcaceae by 4.28%. The genera with an abundance greater than 10% in the control group were *Lactobacillus* and Muribaculaceae, and the genera with an abundance greater than 10% in the infected group were *Bacteroides*, *Lactobacillus*, and Muribaculaceae; the relative abundance of *Escherichia-Shigella* genera increased by 19.71%, *Bacteroides* by 8.07%, and Lachnospiraceae_NK4A136_group by 3.00% compared to the control group increased by 19.71%, *Bacteroides* by 8.07% and Lachnospiraceae_NK4A136_group by 3.00% compared to the control group (Figures 3C,D).

**Figure 4:**
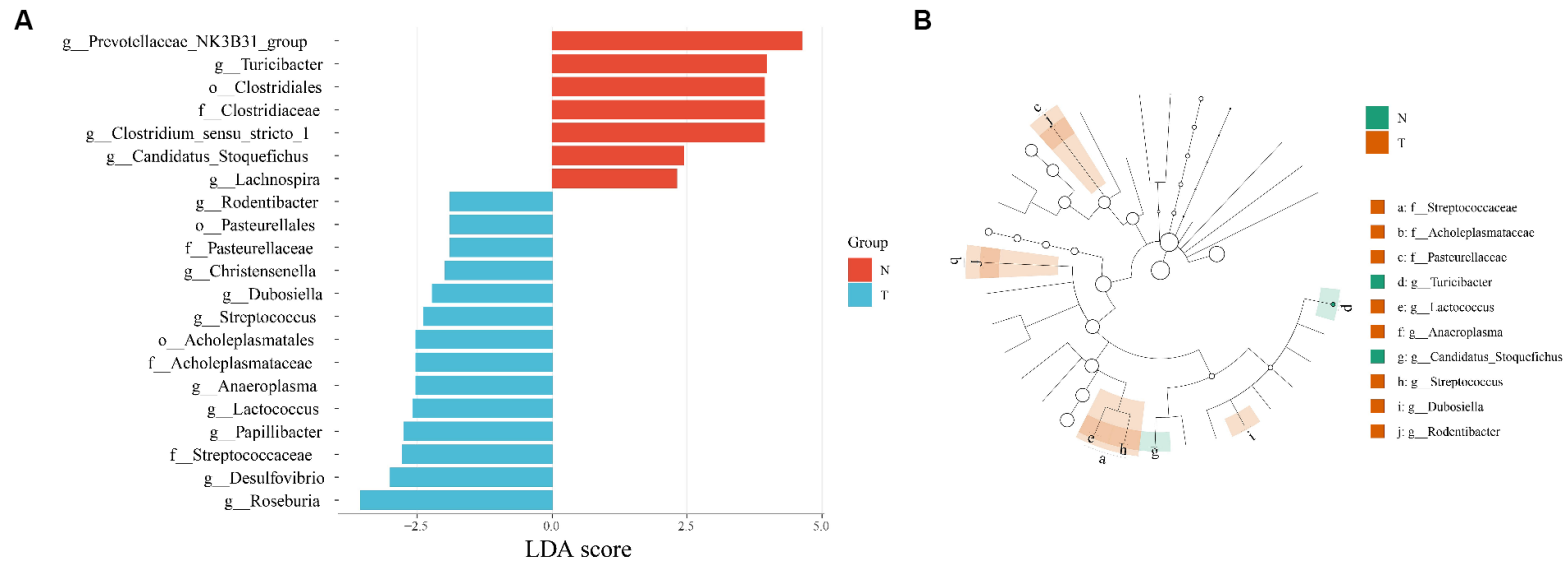
Distribution of LDA values **(A)** and evolutionary branching maps **(B)** of divergent species.

For discovering significant biomarkers between different groups, the LEfSe analysis was performed. The characteristic homogeneous genera in each group were screened and the results are shown in Figure 4A, and the intergroup differences present at each genus level are shown in Figure 4B. The control group contained five characterized genera (Prevotellaceae NK3B31 group, *Turicibacter*, *Clostridium sensu stricto* 1, *Candidatus Stoquefichus*, *Lachnospira*) and the infected group contained nine characterized genera (*Rodentibacter*, *Christensenella*, *Dubosiella*, *Streptococcus*, *Anaeroplasma*, *Lactococcus*, *Papillibacter*, *Desulfovibrio*, *Roseburia*).

### 3.3 Reorganization for intestinal microbiota correlation

**Figure 5:**
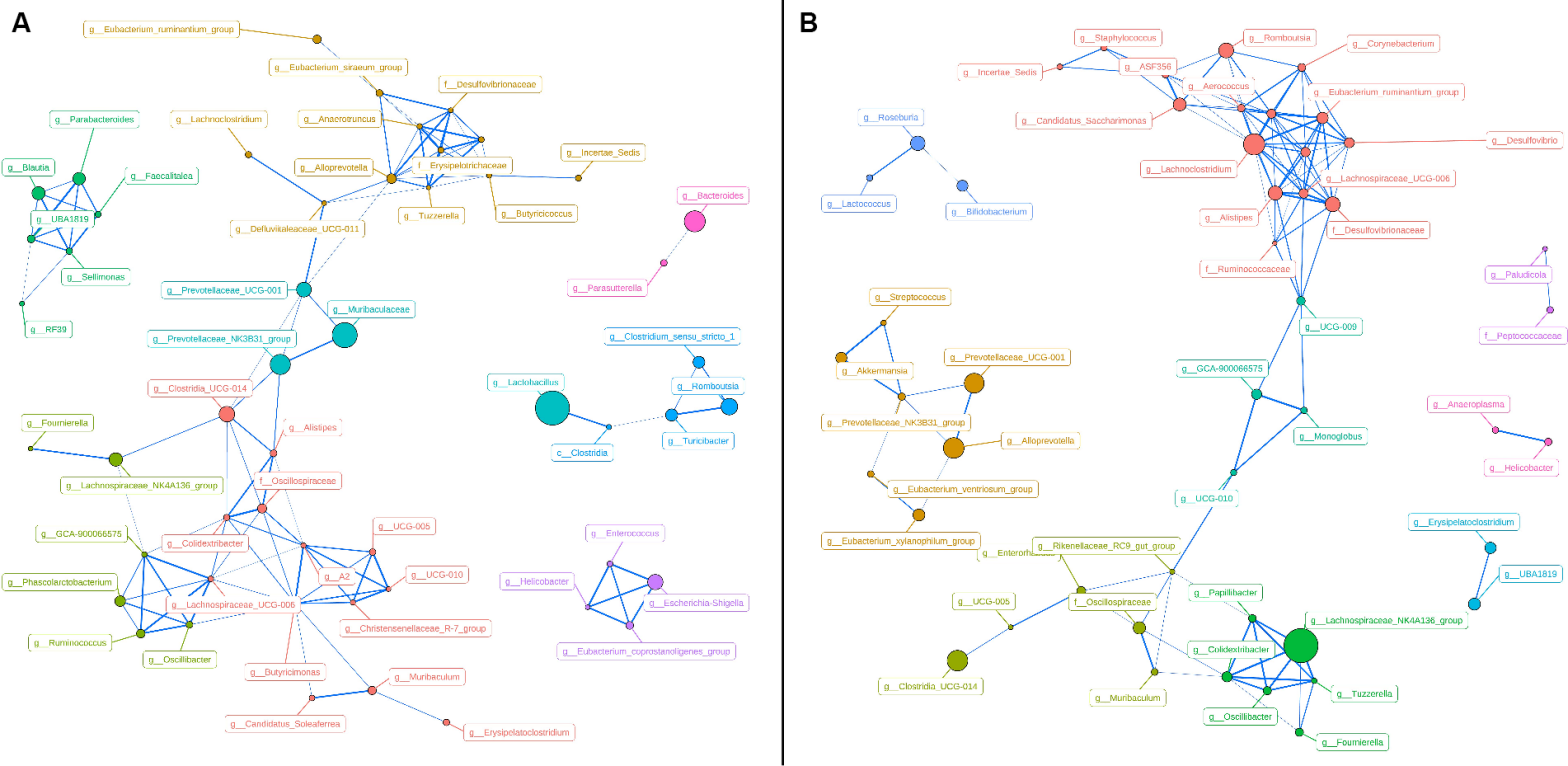
Network analysis chart in control group **(A)** and Infection group **(B)**.

To represent the effect of *A. pegreffii* infection on rat intestinal microbiota, intragroup abundance correlation network diagrams were constructed at the Genus level, respectively. The correlation coefficients were analyzed based on the Spearman method for the 60 most abundant Genera, with the parameters of *R* > 0.6 and *p* < 0.01 (Figure 5). In the control group, the network’ formed two large clusters, while the remaining genera formed smaller connections. The core genera were identified as *Butyricimonas*, *Alloprevotella*, Lachnospiraceae_UCG-006, A2, g_Erysipelotrichaceae, and g_Desulfovibrionaceae based on their weighted degree of centrality. The infection group exhibited two significantly large clusters in the network, which were connected to smaller clusters. The core genera identified were *Lachnoclostridium*, *Eubacterium*_*coprostanoligenes*_group, Lachnospiraceae_UCG-006, and *Alistipes*. Significant changes in abundance and connectivity of differential marker genera in the interactions network are shown in Table 1. Out of all the differential genera, 9 were absent in the network between the control and infection groups, 6 were present only in the interactions network with the control group, and 8 were present only in the interactions network with the infected group. Three genera were present in the interaction network for both the control and infected groups. The *Prevotellaceae*_NK3B31_group had greater weighted centrality in both groups, while *Romboutsia* and *Lachnoclostridium* had greater weighted centrality in the infected group compared to the control group.

**Table 1:** Weighted degree of divergent species with significant changes in the relative abundance.

Species	Weighted degree		Wilcoxon *p*-values
	Control	Infection	
*Romboutsia*	1.943984	5.653698	0.015152
*Lachnoclostridium*	0.967048	12.45731	0.025974
Prevotellaceae_NK3B31_group	4.726978	4.724962	0.002165
RF39	1.848676	-	0.012907
*Butyricicoccus*	6.533377	-	0.02472
Eubacterium_siraeum_group	4.706891	-	0.016711
Escherichia-Shigella	2.963387	-	0.041126
*Turicibacter*	2.844556	-	0.002165
*Clostridium*_sensu_stricto_1	1.897321	-	0.002165
*Desulfovibrio*	-	7.60785	0.009622
*Papillibacter*	-	4.859768	0.002778
*Streptococcus*	-	1.947273	0.007796
f__Peptococcaceae	-	1.883714	0.008658
*Roseburia*	-	1.881372	0.007796
Eubacterium_ventriosum_group	-	1.877816	0.041126
*Anaeroplasma*	-	0.98638	0.002778
*Lactococcus*	-	0.96375	0.004337
*Christensenella*	-	-	0.002671
Candidatus_Stoquefichus	-	-	0.002778
*Dubosiella*	-	-	0.009622
*Anaerostignum*	-	-	0.028441
Christensenellaceae	-	-	0.036379
*Faecalibaculum*	-	-	0.019373
*Lachnospira*	-	-	0.009622
*Rodentibacter*	-	-	0.009622
Erysipelotrichaceae	-	-	0.012592

### 3.4 Functional gene composition of intestinal microbiota in response to *Anisakis pegreffi*

**Figure 6:**
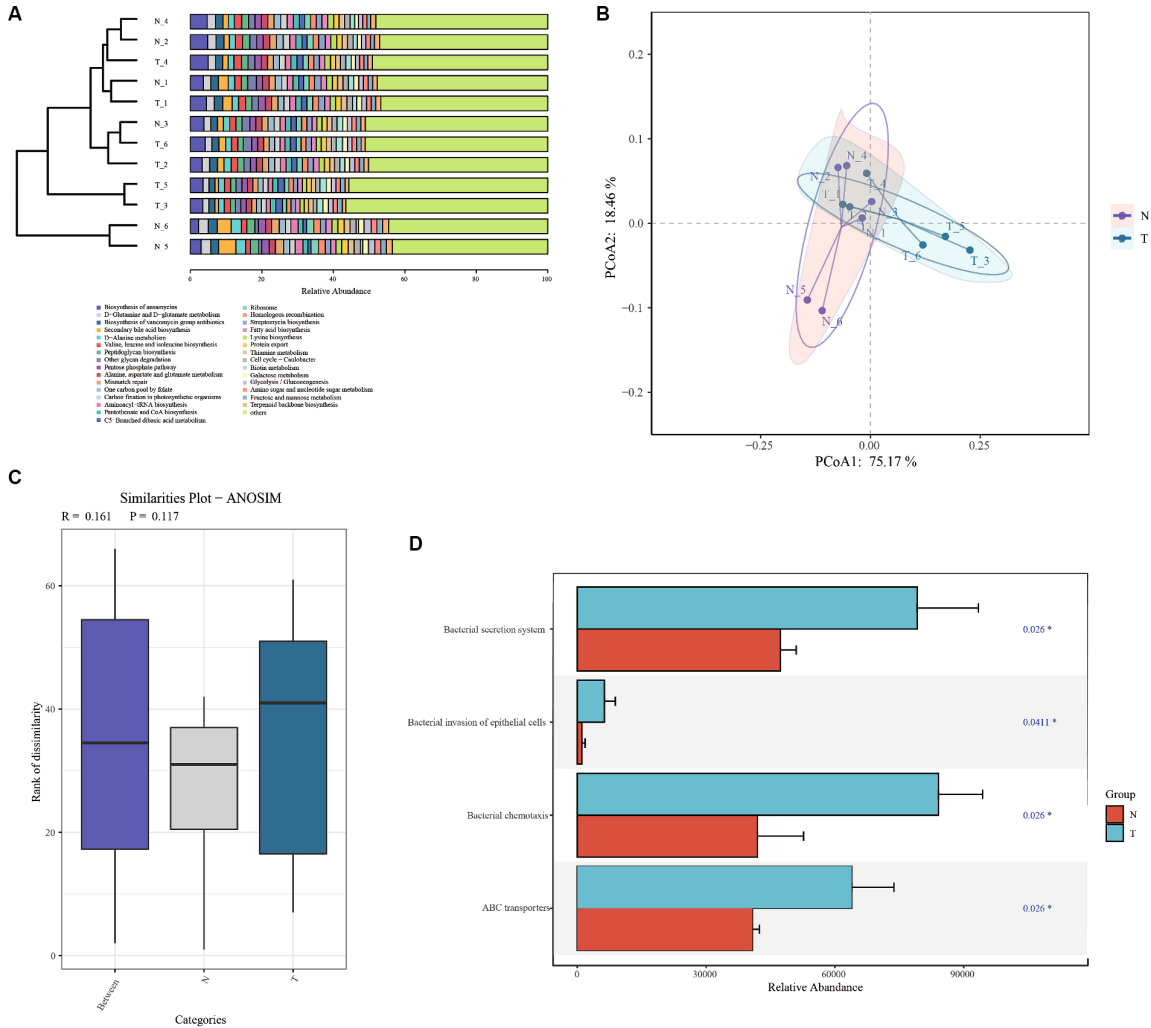
Functional analysis based on PICRUSt predictions. **(A)** Bray Cluster analysis and functional composition stacking map. **(B)** Bray Curtis PCoA analysis plots. **(C)** Anosim analysis based on functional gene abundance. **(D)** Significantly differential functional genes bar chart.

To further research on metabolic differences in microbiota, based on the ASVs and abundances identified by 16S rDNA, the PICRUSt2 tool was used to predict metagenomic functional information (29). Based on the relative abundance of the top 30 functions, the Bray distance was calculated between samples and hierarchical clustering was performed (Figure 6A). The infection group and control group were not clustered together and did not form two independent clusters. The results of the PCoA analysis showed partial overlap between the infected and control groups (Figure 6B). The analysis of the differences between the within and between groups indicated that the between-group difference was larger than the within-group difference (*R* > 0) for the infected and control groups, but it was not significant (*p* > 0.01) (Figure 6C). Based on the Wilcoxon test, four processes were found to be significant: bacterial secretion system, bacterial invasion of epithelial cells, bacterial chemotaxis, and ABC transporters (Figure 6D). All these processes were more abundant in the infection group than in the control group.

## 4 Discussion

Animal models of infection are widely employed to investigate the cause-effect relationships between helminth colonization, shifts in gut microbial composition, and modifications in the immune or metabolic functions of the host (30). However, interactions between helminths and the gut microbiota vary significantly depending on the specific host–parasite combination being studied (31). Consequently, comparing data across different host–parasite systems, even if they involve the same helminth species, could potentially lead to misunderstandings. Conversely, research on naturally infected humans may offer insights into the relationships between gut microbial composition and infection with one or more parasites (32). Moreover, such studies often lack evidence of a causal link between specific gut microbial signatures and susceptibility to or the pathophysiology of infection (33). Therefore, it is important to conduct complementary studies that involve naturally infected human populations in endemic regions alongside experimental animal models to advance our understanding of the role of the gut microbiota in human parasitic diseases (34). The gut microbiota is a crucial element of the immune system and overall health (35). In this study, rats were used to examine the links between *A. pegreffii* infections and the composition of the host gut microbiota. As far as we know, this is the first-ever study investigating the associations between Anisakiasis and the gut microbiota of the host (rats).

Given the recognized risk posed by *Anisakis* nematodes, further investigation into their impact on the host is warranted (36). Our current study found that *A. pegreffii* infection did not lead to significant alterations in the alpha diversity of rat gut microbiota. However, differences in beta diversity were observed, which is in line with previous research (32, 37, 38). Interestingly, we observed a significant reduction in the abundance of the Genus *Turicibacter* and the Order Clostridiales in the infected group, suggesting potential intestinal inflammation (39). This finding is consistent with the results of research that showed a decrease in Clostridiales due to *Toxocara canis* infection. Despite the known ability of Firmicutes bacteria to metabolize free fatty acids and bile acids, resulting in the production of butyrate, the reduced abundance of Firmicutes phylum in our study did not reflect anti-inflammatory properties (40, 41).

The Prevotellaceae_NK3B31_group is a key group of intestinal microorganisms, typically considered for their roles as probiotic bacteria producing short-chain fatty acid (SCFA). They are capable of producing various essential salts and fatty acids from dietary fiber, thereby playing a significant role in animal growth and development. Moreover, an increase in the abundance of the population of this group has been associated with the mitigation of gastric ulcer pathology (42–45). The high weighted degree of centrality of this bacterial group in both the control and infected groups indicates its importance in the intestinal tract of both groups of test animals. However, a significant decrease in the abundance (8% in control groups, 0.1% in infection groups) of this genus in the infection group resulted in a substantial alteration in gut microbiota diversity. Bacteria of the genus *Turicibacter* are also important members of the mammalian gut microbiota with evidence suggesting their involvement in modifying host bile acids and lipids, thereby regulating host lipid metabolism (46, 47). In the infection group, there was a significant decrease in the relative abundance of this genus (2% in control groups, 0.038% in infection groups), leading to a diminished capacity to regulate the overall gut microbiota. Intestinal helminth infections often increase the microbial origin of SCFAs, which can help regulate allergic diseases (48). However, this phenomenon was not observed in the present study. The Genus *Clostridium*_sensu_stricto_1 is known for its production of butyrate, which plays an important role in the development of intestinal epithelial cells and hosts energy supply (49, 50). In the present study, the relative abundance of this genus was significantly reduced (1.9% in control groups, 0.031% in infection groups), along with its ability to regulate the gut microbiota. The decreased level of *Romboutsia* (4.9% in control groups, 0.65% in infection groups) can lead to an increase in intestinal pH, subsequently promoting the proliferation of harmful bacteria like *Streptococcus* (0.007% in control groups, 0.052% in infection groups) (51). In heavily infected individuals with roundworms, *Streptococcus* was found to be the most prevalent bacteria in the intestines, significantly decreasing the pro-inflammatory activity of the host colonic mucosa (52, 53). The metabolism of butyric acid in the host’s intestines has been significantly altered. Indigestible complex carbohydrates, such as dietary fiber, serve as an energy source for specific bacteria, resulting in the production of various short-chain fatty acid molecules, including butyrate. These microbial compounds play a role in regulating various metabolic pathways in the gut and other parts of the body, such as the liver, adipose tissue, muscle, and brain. It’s now well-established that these metabolites have a significant impact on several physiological processes, including energy balance, glucose, and lipid metabolism, inflammation, as well as immunity, and cancer (54–56). Interestingly, the infection group showed enrichment of *Roseburia* (0.015% in control groups, 0.64% in infection groups), typically considered a probiotic that produces butyric acid and contributes to asthma prevention in children (57).

Moreover, the presence of *Lactococcus* was augmented in the infected cohort, aimed at curbing the onset of hypersensitivity reactions (58). The increase in both *Desulfovibrio* (0.043% in control groups, 0.198% in infection groups) and *Streptococcus* may indicate issues with bile acid metabolism, which aligns with the previously mentioned decrease in fatty acid synthesis. The Genus *Desulfovibrio* is commonly regarded as a harmful group of bacteria that produces reduced sulfate, generating endogenous H_2_S that can be detrimental to the organism (59, 60). The relative abundance of *Desulfovibrio* spp. is elevated in the intestinal tract of individuals with colitis, inflammatory bowel disease, and irritable bowel syndrome (61–63). The genus of *Escherichia-Shigella* was more abundant in the infection group (4.104% in control groups, 23.82% in infection groups), which is typically associated with dysentery (64). Additionally, it is positively associated with sulfate toxin metabolism (65). The genus *Bacteroides* was more abundant in the infection group (9.11% in control groups, 17.19% in infection groups) and can produce toxins that cause secretory diarrhea and colonic epithelial damage, leading to chronic colitis and colorectal cancer (66, 67). Bacteria have the potential to harm the host’s health by invading tissue sites and evading the immune system. One of the ways they achieve this is through the secretion of virulent proteins, which can be released into the host cell or surrounding environment (68). Nevertheless, a more in-depth comprehension of the alterations in the intestinal microbiota in infected states can facilitate the treatment by improving the microbiological environment. For instance, the regulation of the composition of gut microbes may be employed to alleviate systemic inflammation and cardiovascular disease caused by chronic nephritis (69). Such research is also frequently employed in the investigation of neurological disorders (70–72).

In this study, based on the analysis of microbiota diversity and functional prediction, the infected group exhibited notable increases in the Bacterial secretion system, invasion of epithelial cells, and ABC transporter functions, implying significant microbiota-induced impact to the host gut.

## 5 Conclusion

In this study, rats infected with *A. pegreffii* were obtained by artificial infection and infection was determined by indirect ELISA diagnosis based on worm extraction. For the first time, the microbiota diversity analysis of host feces was performed using 16S sequencing. The results indicate that rats infected with *A. pegreffii* exhibited significant changes in microbiota diversity. The rats’ SCFAs metabolism was affected, which in turn affected their nutrient metabolism and immune function. The increase in the abundance of pathogenic enteric bacteria indicated that the gut flora had been invaded by pathogens. It provides a basis for future research on host-helminth-microbiota correlations and microbiota metabolism.

## Data availability statement

The datasets presented in this study can be found in online repositories. The names of the repository/repositories and accession number(s) can be found at: https://www.ncbi.nlm.nih.gov/, PRJNA1088275.

## Ethics statement

The animal study was approved by Medical Science and Technology Ethics Committee of Jiujiang University. The study was conducted in accordance with the local legislation and institutional requirements.

## Author contributions

M-hZ: Conceptualization, Data curation, Formal analysis, Investigation, Methodology, Software, Validation, Visualization, Writing – original draft, Writing – review & editing. SL: Methodology, Supervision, Validation, Writing – review & editing. Q-bL: Data curation, Software, Writing – review & editing. X-xW: Conceptualization, Data curation, Investigation, Writing – review & editing. AQ: Investigation, Methodology, Writing – review & editing. MM: Funding acquisition, Resources, Writing – review & editing.

## Footnotes

^1^www.snapgene.com

^2^http://cloudtutu.com.cn/
