# Supplementary material for: Modulation of the rat intestinal microbiota in the course of Anisakis pegreffii infection
Source: Front Vet Sci. 2024 May 9;11:1403920. doi: 10.3389/fvets.2024.1403920 (PMC11111928; doi:10.3389/fvets.2024.1403920)
Supplement: Supplementary file 1 [file Table_1.DOCX]

Supplementary Material

Modulation of the Rat intestinal microbiota in the course of *Anisakis pegreffii* infection

Min-hao Zeng^1*†^, Shan Li^2*†^, Qing-bo Lv^3^, Xiao-xu Wang^1^, Abdul Qadeer^4^, Mohamed H. Mahmoud^5^

^1^School of Biotechnology, Jiangsu University of Science and Technology, Zhenjiang 212100, The People's Republic of China

^2^Precision Preventive Medicine Laboratory of Basic Medical School, Jiujiang University, Jiujiang 332005, Jiangxi, The People's Republic of China

^3^Key Laboratory of Zoonosis Research, Institute of Zoonosis, College of Veterinary Medicine, Jilin University, Ministry of Education, Changchun 130062, The People's Republic of China

^4^Department of Cell Biology, School of Life Sciences, Central South University, Changsha, The People's Republic of China

^5^Department of Biochemistry, College of Science, King Saud University, Riyadh 11451, Kingdom of Saudi Arabia

*** Correspondence:**Min-hao Zeng
[zengminhao@stu.just.edu.cn](mailto:zengminhao@stu.just.edu.cn)

# Supplementary Data

Supplementary Material should be uploaded separately on submission. Please include any supplementary data, figures and/or tables.

Supplementary material is not typeset so please ensure that all information is clearly presented, the appropriate caption is included in the file and not in the manuscript, and that the style conforms to the rest of the article.

# Supplementary Figures and Tables

For more information on Supplementary Material and for details on the different file types accepted, please see [here](https://www.frontiersin.org/guidelines/author-guidelines#supplementary-material).

## Supplementary Figures


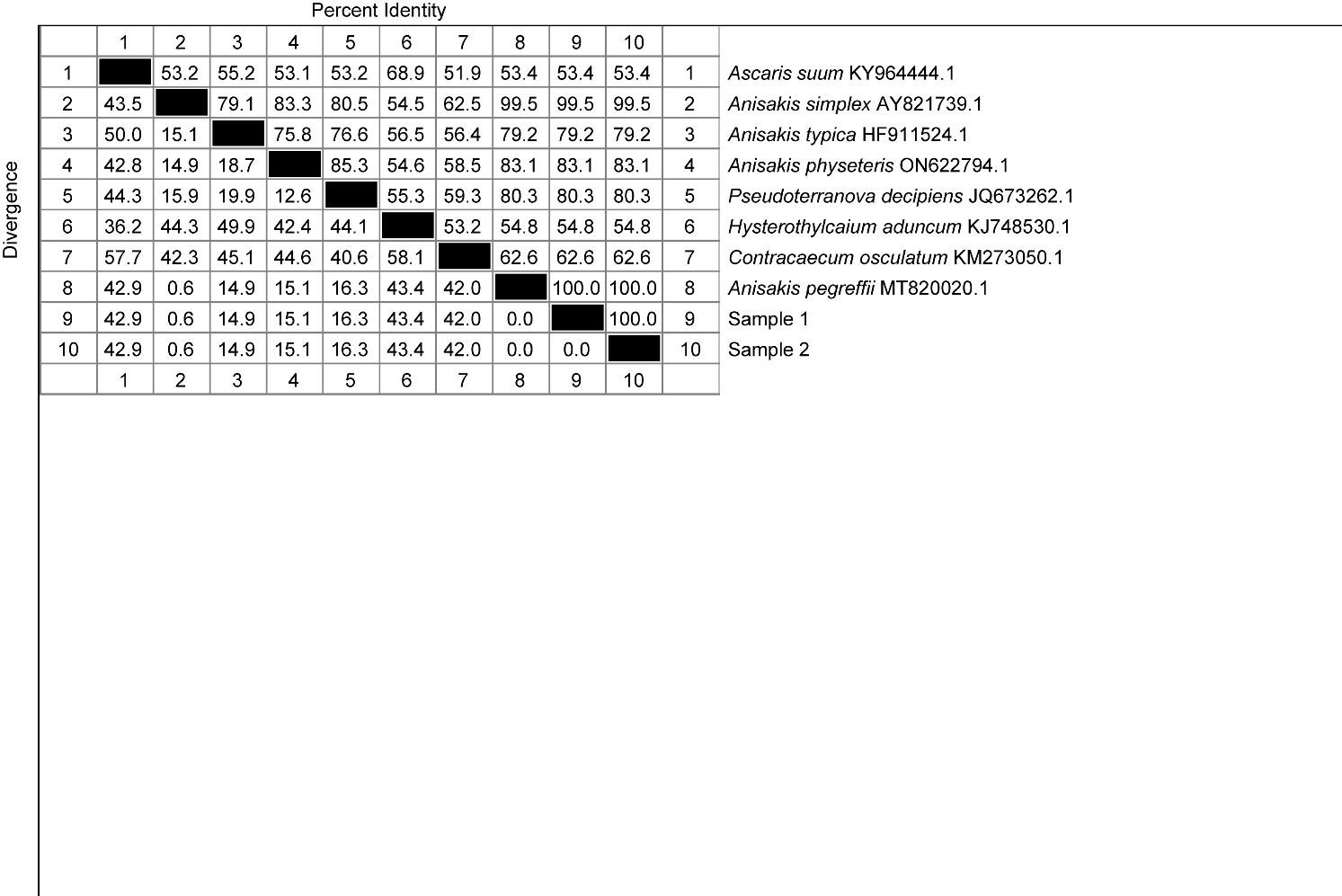


**Supplementary Figure S1.** Divergence of ITS sequences among the nematodes. The two samples were identified as *Ansiakis pegreffii* with the 100% identity.

**
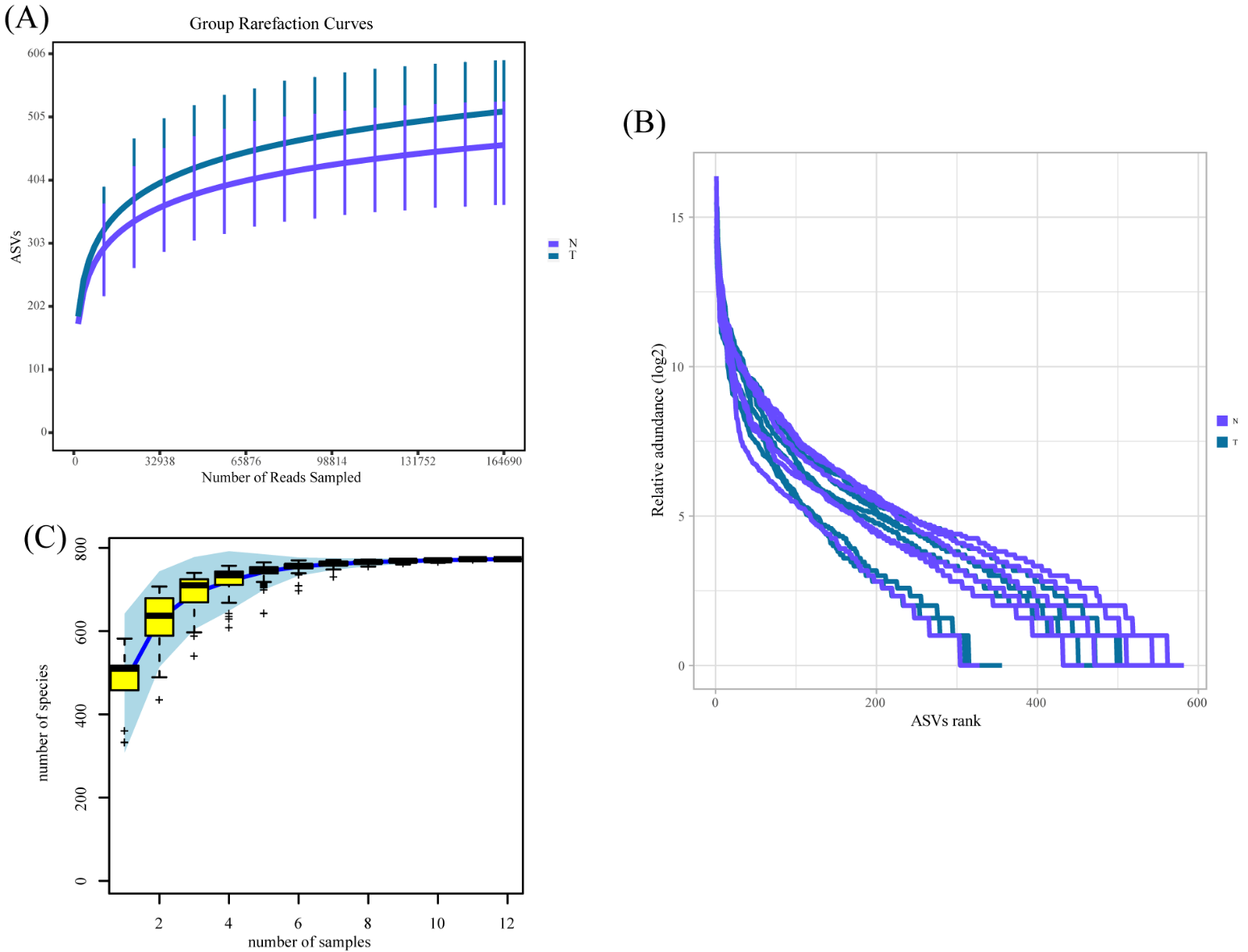
**

**Supplementary Figure S2.** Alpha diversity analysis. (A) Rarefaction curve. As the number of reads increases, the growth curve of ASVs gradually becomes smoother, indicating that the sequencing depth is sufficient. (B) Rank-Abundance curve. The relative abundance curves of the top 200 ASVs are even and smooth, indicating an even distribution of species. (C) Species accumulation curves. With five or more biological replicates, there is no significant increase in the number of species. This indicates that the number of biological replicates in this study meets the analysis requirements.
